# Effects of AH_3_ and AFt on the Hydration–Hardening Properties of the $C_{4}A_{3}\bar{S}$-$C\bar{S}$-H_2_O System

**DOI:** 10.3390/ma16186322

**Published:** 2023-09-21

**Authors:** Xuefeng Li, Songhui Liu, Haibo Zhang, Haiyan Li, Xuemao Guan

**Affiliations:** 1 School of Materials Science and Engineering, Henan Polytechnic University, Jiaozuo 454003, China; 15514076976@163.com (X.L.);; 2 Henan Key Laboratory of Materials on Deep-Earth Engineering, Jiaozuo 454003, China

**Keywords:** calcium sulfoaluminate, AH_3_ phase, AFt, Rietveld quantitative phase analysis, compressive strength

## Abstract

This study aimed to reveal the effects of the hydration products AH_3_ and AFt phases on the hydration and hardening properties of calcium sulfoaluminate (CSA) cement. In addition, the effects of anhydrite ($C\bar{S}$) and gypsum ($C\bar{S}H_{2}$) on the properties of CSA cement were compared. Calcium sulfoaluminate ($C_{4}A_{3}\bar{S})$ was synthesized with analytical reagents, and the $C_{4}A_{3}\bar{S}$-$C\bar{S}$-H_2_O system with different molar ratios of $C\bar{S}$ and $C_{4}A_{3}\bar{S}$ was established. The phase compositions and contents of AFt and AH_3_ were determined by X-ray diffraction (XRD), Rietveld quantitative phase analysis, and thermogravimetric analysis (TG). The effects of pore structure and hydration product morphology on mechanical properties were analyzed by mercury intrusion porosity (MIP) and scanning electron microscopy (SEM). The results showed that the compressive strength exhibited a correlation with the AH_3_ content. In the case of relatively sufficient anhydrite or gypsum, $C_{4}A_{3}\bar{S}$ has a high degree of hydration, and the AH_3_ content can be considered to contribute more to the strength of the hardened cement paste. When anhydrite was selected, the combined and interlocked AFt crystals were covered or wrapped by a large amount of AH_3_. The mechanical properties of the hardened cement paste mixed with anhydrite were better than those of that mixed with gypsum.

## 1. Introduction

Calcium sulphoaluminate (CSA) cement has been widely used in coal mine grouting, marine engineering, and other special construction projects due to its basic properties, such as early strength and early setting [1,2,3]. The sintering temperature for producing CSA cement clinker is usually 1250–1350 °C, which is about 100–200 °C lower than that of OPC cement clinker [4,5,6,7], so it can replace part of OPC cement to reduce CO_2_ emissions [8].

Ye’elimite ($C_{4}A_{3}\bar{S}$), the main mineral of CSA cement, has always been a hot topic of research. The hydration of $C_{4}A_{3}\bar{S}$ is influenced by the addition of calcium sulfate [9,10,11,12]. It has been shown that CSA cement exhibits different properties depending on the type and content of calcium sulfate [13,14,15,16,17,18]. The effects of gypsum and anhydrite on the performance of CSA cement have been studied less. Therefore, the characteristics of different calcium sulfate types and contents on the generation rate, amount, and morphology of the hydration products in CSA cement need to be focused on.

The main hydration products of CSA cement are AFm, AFt, and AH_3_ [19,20,21,22,23]. When $C_{4}A_{3}\bar{S}$ is hydrated, $C_{4}A_{3}\bar{S}$ reacts with water to form AFm and AH_3_ (Equation (1)). With sufficient anhydrite or gypsum content, AFt and AH_3_ can be formed (Equation (2)). According to Equation (3), the amount of anhydrite or gypsum in the system determines the ratio of AFt and AFm in the hydration product, and there is a relationship between the amount of AFt and the AH_3_ phase [24]. It has been shown that the AFt and AH_3_ in the hydration products of CSA cement are related to the strength of hardened cement paste [25,26,27]. F.P. Glasser’s team [28] initially explored the influence of hydration products on the microstructure of hardened cement paste and believed that AFt was generated first, forming a loose network skeleton structure. Song et al. [29] analyzed the microscopic characteristics of the AH_3_ phase in calcium sulfoaluminate–belite cement and found that the AH_3_ phase was encapsulated on the surface of AFt or gypsum. The AFt and AH_3_ phases play pivotal roles in influencing the setting, mechanical performance, and microstructural development of CSA cement systems [30,31]. While previous studies have explored CSA cement hydration and properties, elucidating the specific effects of AFt and AH_3_ quantities on performance remains a critical gap needed to guide further optimization.

(1) $$C_{4}A_{3}\bar{S}+18H\;\to {\;C}_{3}A\cdot C\bar{S}H_{12}(\mathrm{AFm})+2AH_{3}$$

(2) $$C_{4}A_{3}\bar{S}+2C\bar{S}+38H\to C_{3}A\cdot 3C\bar{S}\cdot H_{32}\left(\mathrm{AFt}\right)+2AH_{3}$$

(3) $$C_{4}A_{3}\bar{S}+xC\bar{S}+(18+10x)H\to (1-x/2)\mathrm{AFm}+\left(x/2\right)\left(\mathrm{AFt}\right)+2AH_{3}$$

This study aimed to reveal the linkages between AFt and AH_3_ generation and the resultant properties of CSA cements synthesized with two different calcium sulfate sources, anhydrite and gypsum. By controlling the calcium sulfate type and dosage, tailored $C_{4}A_{3}\bar{S}$-AFt-AH_3_ systems were produced to isolate the impact of these two hydrates. Previous studies have focused on belite–calcium sulfoaluminate cement [32,33,34,35,36,37]. In this paper, Pure $C_{4}A_{3}\bar{S}$ was first synthesized to enable precise control of the calcium sulfate additions without interferences from supplementary cementitious materials. $C_{4}A_{3}\bar{S}$ was hydrated with varying molar ratios of anhydrite or gypsum, and the phase evolutions were quantified using X-ray diffraction and Rietveld analysis. Compressive strength development was measured throughout curing. Mercury intrusion porosimetry and scanning electron microscopy provided insights into pore structure refinement and microstructural densification.

## 2. Experimental Procedure

### 2.1. Preparation of $C_{4}A_{3}\bar{S}$ Clinker

$C_{4}A_{3}\bar{S}$ was synthesized from analytically pure reagents CaCO_3_, Al_2_O_3_, and CaSO_4_ according to the molar ratio (Equation (4)). The analytically pure reagents were mixed together in a pan at the stoichiometric proportion of 3:3:1, and deionized water was added at 10 wt% of the total reagents. This was mixed for 10 min to ensure homogeneity. The mixture was pressed into a cylinder of Φ20 × 2 cm at 10 MPa. The slurry from the wet homogenization first had to be dried completely at 100 °C for 12 h. The raw meal was heated to 900 °C at a ramp rate of 5 °C/min and held for 1 h to precipitate CO_2_ gas. The clinker was heated to 1350 °C [38] at a temperature increase rate of 5 °C/min, held for 3 h. Finally, the clinker was taken out and cooled quickly under the action of a fan. The samples were repeatedly ground in a mortar and sieved through an 80 μm standard sieve and bottled in airtight containers.

(4) $$3{\mathrm{CaCO}}_{3}+3{\mathrm{Al}}_{2}O_{3}+{\mathrm{CaSO}}_{4}\to C_{4}A_{3}\bar{S}+3CO_{2}↑$$

### 2.2. Sample Preparation and Hydration

Controlled $C_{4}A_{3}\bar{S}$-calcium sulfate systems were prepared by hydrating the synthesized $C_{4}A_{3}\bar{S}$ with varying molar ratios of anhydrite ($C\bar{S}$) or gypsum ($C\bar{S}H_{2}$), as listed in Table 1. The calcium sulfate molar ratio x [39] ranged from 0 to 3 according to Equation (2), where x = 0 represents $C_{4}A_{3}\bar{S}$ hydration without any addition. The corresponding mass ratios of $C_{4}A_{3}\bar{S}$ to $C\bar{S}$/$C\bar{S}H_{2}$ were calculated based on the molar masses. Pastes were prepared with a water–cement ratio of 0.8 and cast into 20 mm cube molds. The hydration kinetics of cubic $C_{4}A_{3}\bar{S}$ strongly depend on the w/s ratio [40]. The water–cement ratio satisfies the theoretical water requirement for all experiments [41] and ensures that there is no leakage during the preparation of the slurry. Samples were demolded after 1 day of curing at 20 °C and 50% relative humidity, then tested at ages up to 28 days. Compressive strength was measured on triplicate cubes at each age. Hydration was halted on fractured portions by ethanol immersion before phase and microstructural characterization.

### 2.3. XRD and Rietveld Quantitative Phase Analysis

The hydrated samples were stopped by immersing in alcohol for 24 h. The hydrated samples were dried by using a vacuum drying box. The temperature and the vacuum degree were 35 °C and 0.08 MPa, respectively. Finally, the clinkers were ground to pass through an 80 μm sieve.

XRD was conducted over 10–80° 2θ using a Smart-Lab X-ray diffractometer (Rigaku, Japan, Cu target) with Cu Kα radiation to identify the crystalline phase assemblages. Rietveld quantitative phase analysis was performed to determine the amounts of reactant and hydrated phases [42,43,44,45,46]. An amount equal to 10 wt% ZnO was added as an internal standard for quantifying amorphous content, including AH_3_. Refined parameters included lattice constants, atomic positions, peak shapes, preferred orientations, and phase fractions. The amorphous content was calculated by the difference between the total and crystalline mass fractions.

### 2.4. TGA––DTG Test

A comprehensive thermal analyzer HCT-3 (Beijing Hengjiu Scientific Instrument Factory, Beijing, China) was used for the thermogravimetric analysis (TGA) test. Before the analysis, the sample powder (15–20 mg) was placed in a corundum crucible and tested at a heating rate of 10 °C/min and the atmosphere was air.

### 2.5. MIP Test

Mercury intrusion porosimetry (MIP) was performed using an automatic mercury pore meter, AutoPore IV 9510, supplied by Micromeritics Co., Ltd. (Norcross, GA, USA). According to the volume of the sample tube, a sample mass of about 3.5 g is suitable. Before the test, the samples were immersed in an excess of anhydrous ethanol for 24 h to stop the hydration reaction. The samples were then dried in a vacuum desiccator at 35 °C for 48 h. During the test, the preparation method of the test block was consistent, and the size was uniform.

### 2.6. SEM Test

The samples were dried and made into blocks smaller than 5 mm, and the surface was sprayed with gold to make the surface conductive. The microstructural characteristics of the samples were observed using a scanning electron microscope (Merlin Compact, Carl Zeiss NTS GmbH, Oberkochen, Germany) at 15 kV. The working distance range was 0.1–50 mm.

## 3. Results and Discussion

### 3.1. Phase Composition and Particle Size Distribution of Clinker

**Figure 1:**
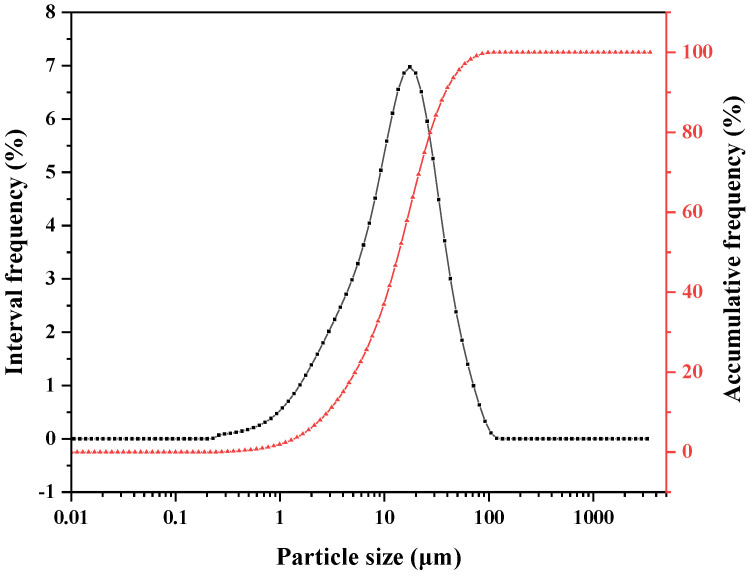
Particle size distribution of clinker.

**Figure 2:**
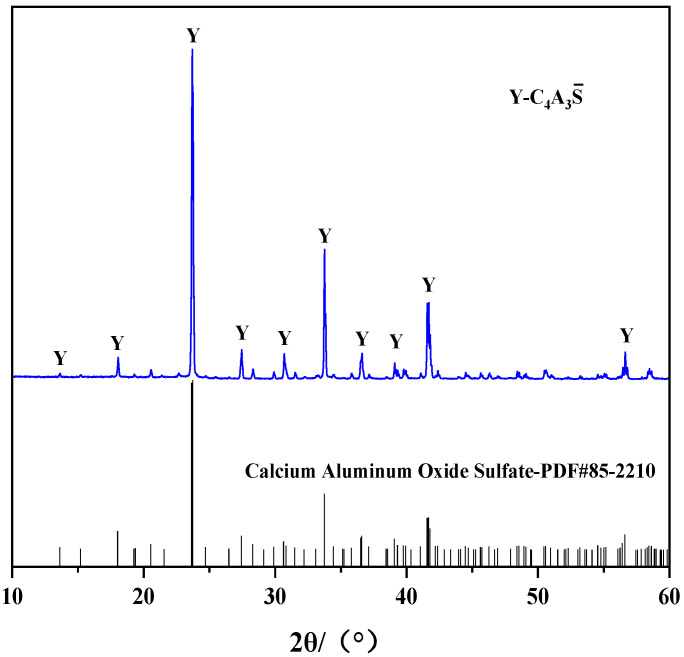
XRD pattern of $C_{4}A_{3}\bar{S}$.

Figure 1 shows the particle size distribution curves of the clinker. The values of d10, d50, and d90 are 2.8, 13.7, and 38.3 μm, respectively. An XRD test was performed to obtain the physical phase composition of the clinker, as shown in Figure 2. Through the comparison of the PDF card, the clinker phase was pure $C_{4}A_{3}\bar{S}$.

### 3.2. Compressive Strength Tests

**Figure 3:**
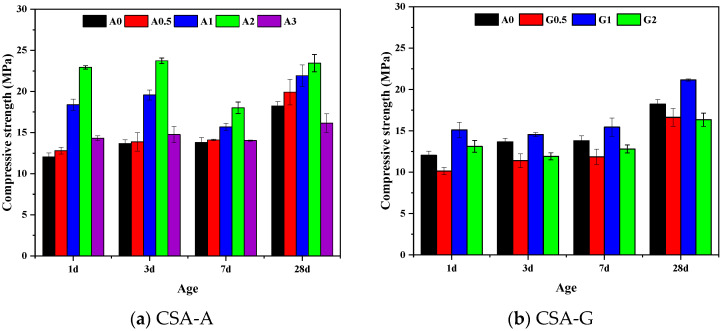
Compressive strength results of $C_{4}A_{3}\bar{S}\;$ hydrated samples.

One of the basic characteristics of CSA cement is its high early strength. The compressive strength of the CSA-A and CSA-G samples are presented in Figure 3. It can be seen from Figure 3a that the compressive strengths of A0 and A0.5 are similar at around 1–7 days, with the highest compressive strength until 28 days of curing. With the increase in anhydrite content, the compressive strength of the samples increases first and then decreases. When the molar ratio of anhydrite and $C_{4}A_{3}\bar{S}$ is 2, the compressive strength of A2 is the highest, and the compressive strength of the A3 sample decreases sharply. This is mainly due to an excess of anhydrite, and a reduction in $C_{4}A_{3}\bar{S}$ will result in a loss of strength.

Figure 3b shows that at the same hydration age, the compressive strength is G1 > A0 > G2 > G0.5. Too little or too much gypsum content will adversely affect the mechanical properties of CSA cement. The strengths of the samples at the ages of 1–7 days are relatively close, and the mechanical properties of the G1 sample are the best. The increase in gypsum in G2 reduces the content of $C_{4}A_{3}\bar{S}$ in the system, resulting in a decrease in the strength of the sample. Comprehensively comparing the strength of each age, the compressive strength of CSA-A is greater than that of CSA-G.

### 3.3. XRD and Rietveld Quantitative Phase Analysis

**Figure 4:**
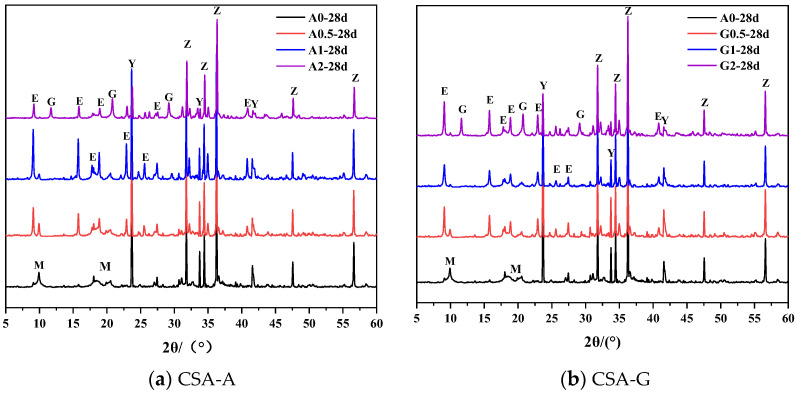
XRD patterns of $C_{4}A_{3}\bar{S}$ hydrated samples at 28 days. Legend: Y: $C_{4}A_{3}\bar{S}$, E: AFt, M: AFm, G: Gypsum, Z: ZnO.

Figure 4 shows that the peak height variation of AFt is closely related to the content of anhydrite and gypsum. The PDF cards used for the analysis are AFt (PDF#71-0969), AFm (PDF#83-1289), Gypsum (PDF#33-0311), and ZnO (PDF#70-2551). The hydration of the A0 sample produces a small amount of AFt and a large amount of AFm. The hydration of pure $C_{4}A_{3}\bar{S}$ can produce ettringite without external SO_4_^2−^ sources [15], which may be due to the involvement of free SO_4_^2−^ during the dissolution of the mineral. It is observed that a large amount of AFt and a small amount of AFm are formed in A0.5 and G0.5. The hydration effect of calcium sulfoaluminate is significantly different in the presence and absence of calcium sulfate [47]. Continuing to increase the anhydrite content, only AFt diffraction peaks were available in the A1 and A2 samples.

It is noteworthy that anhydrite is hydrated to gypsum in A2, and excess gypsum remains in G2. Compared with that in A2, the diffraction peak intensity of AFt in A1 is higher, but the compressive strength of the A2 sample is higher than that of A1, which may be related to the content of AH_3_ in the hydration product. Regardless of the amount of added anhydrite or gypsum, the diffraction peaks of $C_{4}A_{3}\bar{S}$ always exist during the hydration process.

The amount of AFt is affected by the reactivity of calcium sulfate (solubility and dissolution rate) at early ages [15]. Due to the faster dissolution of sulfate ions in gypsum than in anhydrite, the formation of AFt generated by hydration is faster, resulting in a weaker AFm diffraction peak intensity in G0.5 than in A0.5. The rapid dissolution of gypsum enables the faster formation of AFt and the formation of a dense matrix based on small crystals. When anhydrite is used, the slow release of sulfate ions affects the formation kinetics of AFt. On the other hand, a better crystalline structure is produced, and this part will be further explained in the subsequent SEM analysis. This explains that the compressive strength of CSA-A is greater than that of CSA-G.

**Figure 5:**
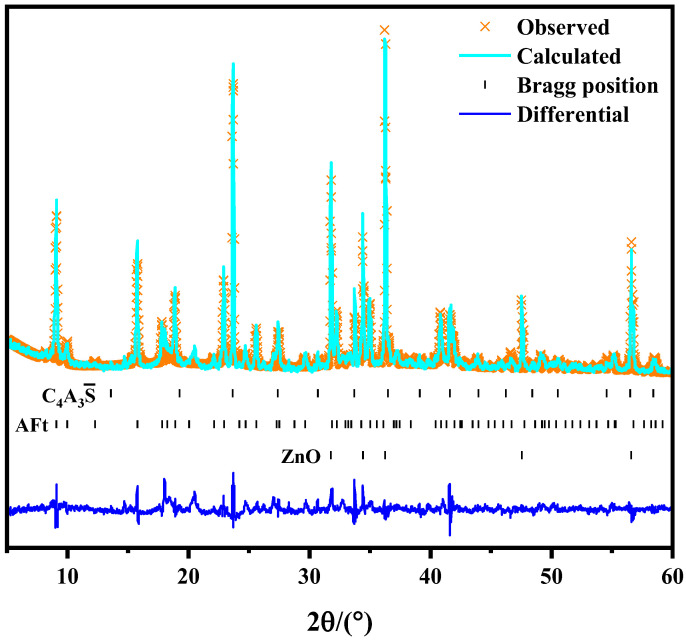
The Rietveld plots for the hydrated sample of A1-28d.

The Rietvel quantitative analysis method has been used to determine the content of all the hydration products in the samples. This part of the study was to quantify the effects of the main hydration products AFt and AH_3_ in the hydration system on the mechanical properties of the hardened cement paste. The quantitative result of A1-28d is chosen in Figure 5. The results of the quantitative analysis of some samples are shown in Table 2. The fitting results show that the Rwp values were all lower than 15%, indicating that the detection of phase composition is very accurate.

**Figure 6:**
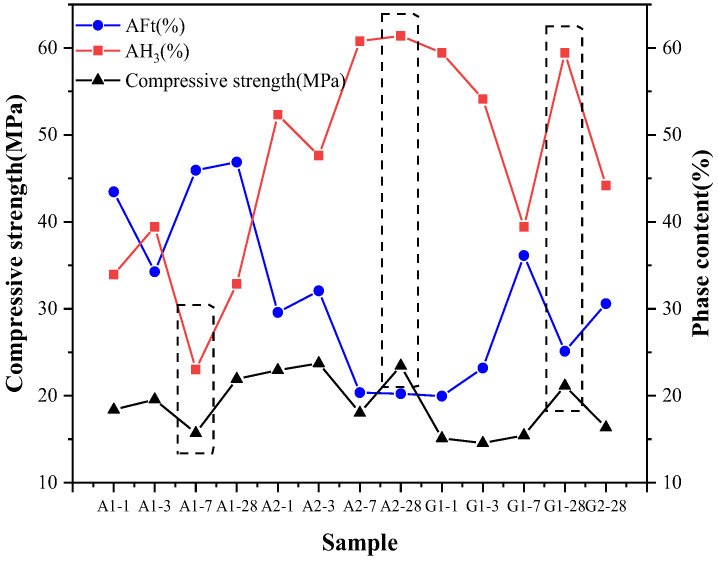
AFt content, AH_3_ content, and compressive strength of different samples.

Figure 6 shows the variations of AFt content, AH_3_ content, and compressive strength of different samples. It can be seen that the trend of AFt content is opposite to that of AH_3_ content, while the trend of compressive strength is the same as that of AH_3_. The compressive strengths of samples A1-7d, A2-28d and G1-28d show obvious correlation with the trend of AH_3_ content as indicated by the dashed box markers in the figure. The compressive strength of A2 was higher than that of A1 due to the higher hydration of $C_{4}A_{3}\bar{S}$ and more AH_3_ content at the same age.

**Figure 7:**
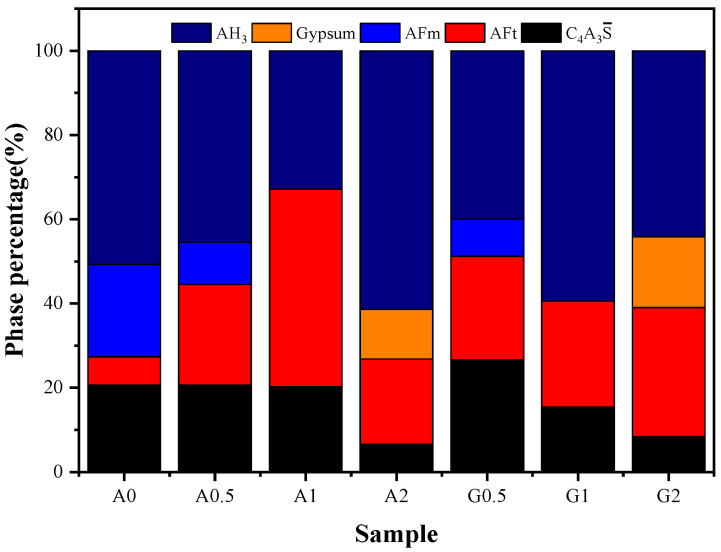
Percentage mass content of hydrated cement pastes at 28 days.

Figure 7 shows the percentage mass content of hydrated cement pastes at 28 days. With the addition of anhydrite and gypsum, the degree of hydration of $C_{4}A_{3}\bar{S}$ increases. Comparing A1 and A2, G1 and G2 found that A2 and G1, with more AH_3_ content, have higher compressive strength. However, not all samples have higher compressive strength with larger AH_3_. The content of AH_3_ in A1 is less than that in A0 and A0.5, but the compressive strength is higher, mainly because the content of AFt in A1 is the highest. The degree of hydration of $C_{4}A_{3}\bar{S}$ in G0.5-28d is 43.2%, which is much lower than other samples. Therefore, its compressive strength is lower than that of other groups of samples.

Only in the case of relatively sufficient anhydrite or gypsum, the hydration of $C_{4}A_{3}\bar{S}$ is more complete, and the AFt in the hydration product is close to complete generation. The AFt crystals overlap each other to function as a backbone and AH_3_ fills the voids. The more AH_3_ is generated, the higher the density of the hardened cement paste. At this time, the amount of AH_3_ generated can be considered to have a greater contribution to the strength of the hardened body. The amorphous phase is generally a gel with a large specific surface area, in which the specific surface area of AH_3_ can reach 285 m^2^/g [48], and the filling of AH_3_ in the hardened cement paste can play a stronger structural support role.

### 3.4. TGA–DTG of the $C_{4}A_{3}\bar{S}$ Hydration System

**Figure 8:**
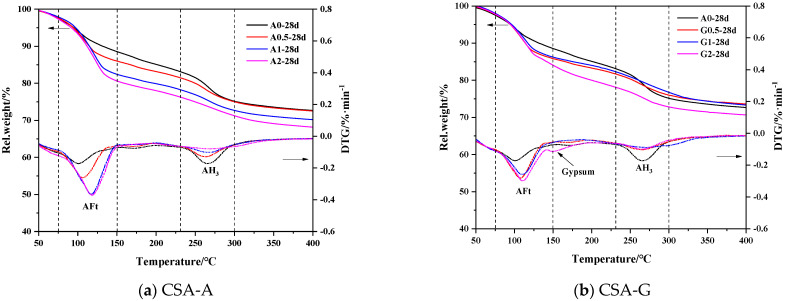
TG–DTG curves of $C_{4}A_{3}\bar{S}$ hydrated paste.

Three distinct mass loss peaks can be observed in Figure 8. Related literature indicates that AFt usually decomposes in the temperature range of 100–150 °C [29]. The main exothermic peak of water loss in AFm is around 180 °C [49] and the dehydration of gypsum occurs around 150 °C. The amorphous AH_3_ phase, with its bound water, decomposes in the temperature range of 250–280 °C [50]. Considering the actual DTG curve analysis for this experiment to delineate, AFt dehydrates at 120 °C with heat absorption and mass loss in the temperature range of 75–150 °C. AFm has a typical heat absorption peak at 180 °C with mass loss in the temperature range of 155–200 °C and AH_3_ has a significant heat absorption peak with mass loss at 230–300 °C.

The A0 sample was more pronounced in the dehydration and weight loss peaks of AFm and AH_3_ than the A1 and A2 samples. Although the A0 sample has higher AH_3_ content, its AFt content is much lower than that of the sample with anhydrite, which also led to its lower strength when compared to the other samples. Comprehensively comparing the AFt peaks of each age, the AFt content of CSA-A is generally larger than that of CSA-G, which is consistent with the quantitative XRD analysis. The DTG curve of G2-28d has a small peak at 150 °C, which indicates the existence of gypsum. According to the quantitative analysis of XRD, the excess of gypsum in G2 is 16.80%.

### 3.5. MIP Results

**Figure 9:**
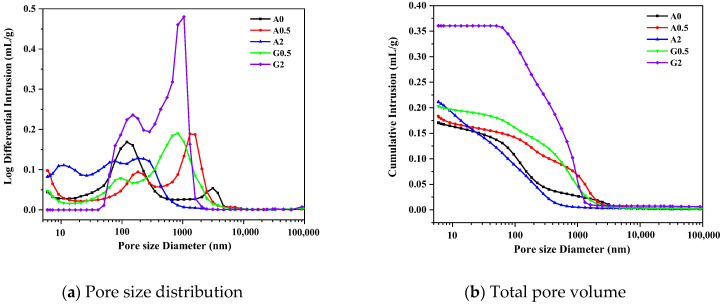
Pore structure of $C_{4}A_{3}\bar{S}$ hydrated paste at 28 days.

Figure 9 shows the pore size distribution curves of different samples at 28 days. Table 3 shows the total pore area, total intrusion volume, average pore diameter, median pore diameter, and pore distribution of the samples. The A0.5, G0.5, and G2 samples have two peak pore sizes in the 10–10,000 nm pore size range, with the highest peaks corresponding to 120.78 nm, 1311.40 nm, 830.27 nm, and 1045.88 nm, representing the highest concentration of pores in the sample.

The average and median pore sizes of A2 are the smallest, and the total pore area is also the largest at 28.589 m^2^/g, indicating that there are more small pores, and the average pore size is small. Comparing the pore size ratios in different pore size ranges, it is found that there are more small pores in CSA-A and more large pores in CSA-G. The G2 sample is especially obvious, and the pore size larger than 100 nm accounts for 91.16%, which confirms that the compressive strength of the G2 sample at 28 days is lower than that of the other groups of samples.

The average pore size of CSA-A is smaller, and the total pore area is larger, compared with that of CSA-G. Combined with the compressive strength analysis of hardened cement paste, the overall compressive strength of CSA-A is higher than that of CSA-G. The results of the MIP testing showed a good correlation with the mechanical properties of the samples.

### 3.6. SEM Analysis

**Figure 10:**
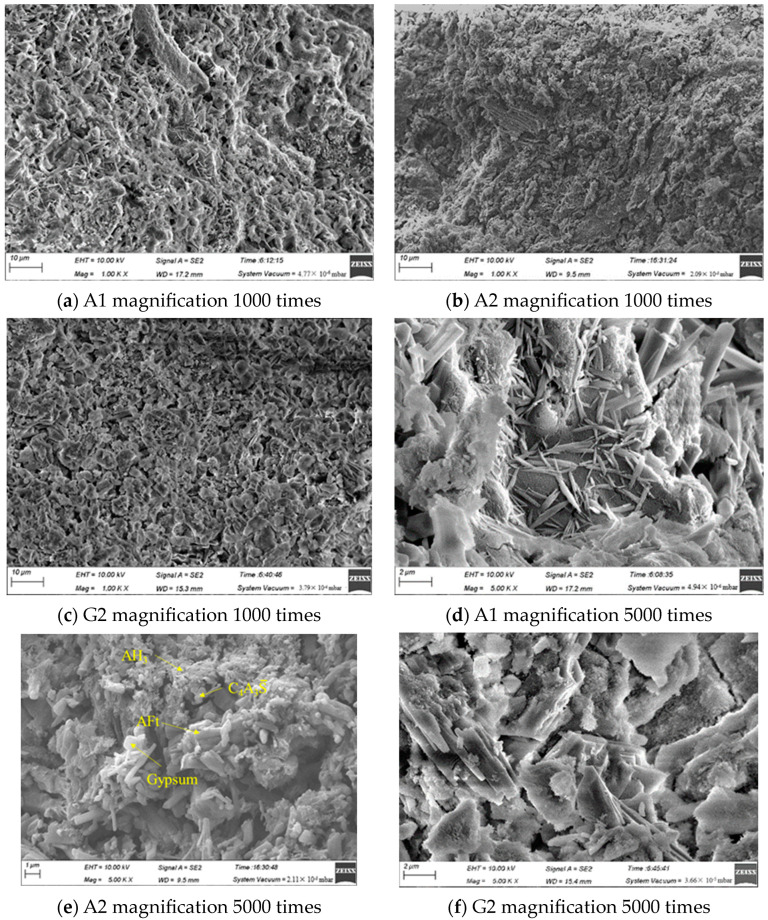
SEM images of the hydrated samples at 28 days.

Figure 10 shows the SEM images of the hydrated samples cured for 28 days. It can be seen that the hydration products include cement matrix, $C_{4}A_{3}\bar{S}$, columnar and acicular AFt, and villous AH_3_. From Figure 10a–c after magnification 1000 times, it can be seen that A2 is denser than A1 and G2 in terms of lap degree, with a denser matrix and fewer voids. The lower porosity of CSA-A compared to CSA-G for the same molar ratio of admixture determines that A2 has higher compressive strength.

It is observed in Figure 10d–f that the columnar AFt in A1 is well crystallized (the part of the AFt of 1 μm in width and 6 μm in length). The bonding and interlocking of adjacent AFt crystals can be seen in the A2 sample, while a large amount of AH_3_ covers or wraps around the AFt. The structural space of the sample can be effectively filled, so the A2 sample has many small pores with a small average pore size. Partially hydrated columnar gypsum crystals can be seen in A2, which are formed by the hydration of unreacted anhydrite. In G2, the cement matrix grows in layers, and the degree of binding and encapsulation of the AFt and AH_3_ is weak.

The dissolution rate of gypsum is larger than that of anhydrite, and AFt tends to grow around the clinker particles during hydration, which reduces the bridging effect of AFt and weakens the degree of binding between AFt and AH_3_ [51]. Anhydrite’s slower dissolution produced superior AFt crystallization with interlocked columnar crystals, enabling enhanced AH_3_ formation in these confined regions. Once sufficient calcium sulphate was present, the AH_3_ content increased, and the pore structure became finer and denser.

## 4. Conclusions

The results of this study provide new insights into the effects of AFt and AH_3_ on the hydration and strength development of CSA cements synthesized with different calcium sulfate sources. The trends in AFt and AH_3_ contents as a function of calcium sulfate type and dosage elucidate their relative contributions to properties and performance.

Several key findings emerged from the measured phase quantities, pore structures, microstructures, and compressive strength. First, sufficient calcium sulfate addition is required to enable high $C_{4}A_{3}\bar{S}$ hydration, without which the AFt and AH_3_ generation is limited. This highlights the importance of calcium sulfate as a hydration moderator in these systems. Once adequate calcium sulfate was present, the AH_3_ content exhibited a direct correlation with strength, while AFt displayed an inverse trend. As AH_3_ increased with greater calcium sulfate up to x = 2, the pore structures became finer and denser. Thus, AH_3_ appears to play a pivotal role in densification and strength development when the $C_{4}A_{3}\bar{S}$ hydration is high.

Notably, anhydrite outperformed gypsum at the same molar ratios, which is ascribed to the different sulfate ion release rates. Anhydrite’s slower dissolution produced superior AFt crystallization with interlocked columnar crystals, enabling enhanced AH_3_ formation in these confined regions. In contrast, gypsum promoted more disordered AFt and porous microstructures. These findings highlight the importance of calcium sulfate selection in addition to dosage for optimizing performance.

Overall, this study clarifies the linkages between AFt and AH_3_ generation and the resultant CSA cement properties. Controlling the calcium sulfate source and content enables tailored hydrated phase assemblages to engineer enhanced mechanical properties, setting the stage for further optimization of these sustainable cement formulations.

## Figures and Tables

**Table 1:** Sample designations, calcium sulfate types and contents.

Sample	x *	$C_{4}A_{3}\bar{S}$$/C\bar{S}(C\bar{S}H_{2}$) Molar Ratio	$C_{4}A_{3}\bar{S}$$/C\bar{S}(C\bar{S}H_{2})$ Mass Ratio	W/C Ratio	Calcium Sulfate
A0	0	1:0	100/0	0.8	-
A0.5	0.5	1:0.5	89.97/10.03	0.8	Anhydrite (CSA-A)
A1	1	1:1	81.77/18.23	0.8	
A2	2	1:2	69.16/30.84	0.8	
A3	3	1:3	59.92/40.08	0.8	
G0.5	0.5	1:0.5	87.64/12.36	0.8	Gypsum (CSA-G)
G1	1	1:1	78.01/21.99	0.8	
G2	2	1:2	63.94/36.06	0.8	

* Notes: x represents the molar ratio of $C_{4}A_{3}\bar{S}$/$C\bar{S}(C\bar{S}H_{2})$.

**Table 2:** Rietveld quantitative analysis results of different samples.

Sample	$C_{4}A_{3}\bar{S}$ (%)	AFt (%)	AFm (%)	Anhydrite (%)	Gypsum (%)	AH_3_ (%)	Compressive Strength (MPa)	$\mathrm{Hydration}\;\mathrm{Degree}\;\mathrm{of}\;C_{4}A_{3}\bar{S}$ (%)	R_wp_
A1-1d	22.6	43.4	0.0	0.0	0.0	34.0	18.4	50.2	12.7
A1-3d	26.3	34.3	0.0	0.0	0.0	39.4	19.6	42.1	12.6
A1-7d	26.0	45.9	0.0	0.0	5.0	23.0	15.7	42.7	11.4
A1-28d	20.3	46.9	0.0	0.0	0.0	32.9	21.9	55.4	10.2
A2-1d	11.5	29.6	0.0	3.6	2.9	52.3	22.9	70.0	8.1
A2-3d	12.0	32.1	0.0	6.0	2.4	47.6	23.7	68.8	11.2
A2-7d	7.9	20.4	0.0	0.0	10.9	60.8	18.0	79.3	8.6
A2-28d	6.6	20.2	0.0	0.0	11.8	61.4	23.5	82.8	9.0
G1-1d	20.6	20.0	0.0	0.0	0.0	59.4	15.1	52.4	10.1
G1-3d	22.7	23.2	0.0	0.0	0.0	54.1	14.6	47.7	11.0
G1-7d	24.4	36.1	0.0	0.0	0.0	39.4	15.5	43.6	11.2
G1-28d	15.5	25.1	0.0	0.0	0.0	59.4	21.2	64.3	10.7
G2-28d	8.4	30.6	0.0	0.0	16.8	44.2	16.3	76.3	8.2
A0-28d	20.7	6.7	21.9	0.0	0.0	50.7	18.2	62.8	9.9
A0.5-28d	20.6	23.9	10.1	0.0	0.0	45.4	19.9	58.7	10.8
G0.5-28d	26.6	24.6	8.9	0.0	0.0	39.9	16.6	43.2	11.6

**Table 3:** Pore structure parameters of $C_{4}A_{3}\bar{S}$ hydrated pastes at 28 days.

Sample	Total Pore Area (m^2^/g)	Total Intrusion Volume (cm^3^/g)	Average Pore Diameter (4 V/A) (nm)	Median Pore Diameter (Volume) (nm)	Pore Distribution/%			
					Less than 10 (nm)	10–50 (nm)	50–100 (nm)	Greater than 100 (nm)
A0	12.206	0.1709	56.02	131.40	5.11	14.61	16.58	63.70
A0.5	12.981	0.1834	56.52	496.76	8.28	8.53	5.29	77.90
A2	28.589	0.2116	29.60	69.50	12.64	29.79	15.38	42.19
G0.5	9.255	0.2032	87.82	488.74	3.90	7.40	8.75	79.95
G2	5.672	0.3605	254.19	460.31	0.00	0.00	8.84	91.16

## Data Availability

The raw/processed data required to reproduce these findings cannot be shared at this time as the data also forms part of an ongoing study.
